# An open dataset for individual tree detection in UAV LiDAR point clouds and RGB orthophotos in dense mixed forests

**DOI:** 10.1038/s41598-024-72669-5

**Published:** 2024-09-20

**Authors:** Ivan Dubrovin, Clement Fortin, Alexander Kedrov

**Affiliations:** 1 Skolkovo Institute of Science and Technology, Center for Digital Engineering, Moscow, Russia; 2 Space Technologies and Services Center, Ltd, Perm, Russia

**Keywords:** Individual tree detection (ITD), UAV LiDAR, RGB, Open dataset, Forestry, Scientific data

## Abstract

We present an open access dataset for development, evaluation, and comparison of algorithms for individual tree detection in dense mixed forests. The dataset consists of a detailed field inventory and overlapping UAV LiDAR and RGB orthophoto, which make it possible to develop algorithms that fuse multimodal data to improve detection results. Along with the dataset, we describe and implement a basic local maxima filtering baseline and an algorithm for automatically matching detection results to the ground truth trees for detection algorithm evaluation.

## Introduction

Forests are an essential renewable natural resource and an important dynamic part of the global carbon cycle. Responsible forest management allows for efficient use of it as a resource and regulation of atmospheric $\text {CO}_2$ but requires up-to-date data about forest attributes such as distribution of species, above-ground biomass, age and height of the trees, and others^1,2^. This drives the need for the ability to quickly and accurately map forest attributes, often covering incredibly large areas, which makes manual forest inventories too costly and time-consuming for extensive, repeated monitoring. LiDAR has been used to augment and extrapolate limited field measurements to larger areas for a long time^3^. It is an active sensor, which means it does not depend on environmental conditions such as lighting, which greatly affects passive sensors like cameras and greatly reduces the universality of methods developed for them. It also penetrates the canopy and provides information about the vertical structure of the forest, including the shape of the terrain and lower levels of trees. UAV LiDAR, out of all other ways to collect LiDAR data, such as from planes or from the ground, is relatively accessible and allows for most configuration of attribution parameters, with a good balance of data quality, cost, and effort.

The most common way to use LiDAR for mapping forests in industry is the area-based approach. It consists of calculating aggregated point cloud metrics for available ground plots, fitting statistical models to predict required attributes, and applying these models on wall-to-wall metrics to extrapolate the measurements^4^. Area-based approach can work with low density LiDAR point clouds, is easy to understand, implement, and extend with other data sources, but the spatial resolution of the results is very coarse. In contrast, modern sensors allow for much more detailed measurements which make it possible to not aggregate at all, instead working on the scale of individual trees. A lot of research is currently being done into the development of methods for detecting individual trees in point clouds, focusing especially on complex forest types^5–7^. Similar problems are solved relatively easily in forests that are either predominantly coniferous or sparse, since both have canopy structures that are easily interpretable, and tree detection can be done using simple algorithms such as local maxima filtering. Forests that are dense and mixed have canopy structures that are a lot more complex and require more sophisticated algorithms and additional data sources to process. The canopies of broadleaf trees rarely have simple shapes with a single height maximum in the middle, rendering local maxima algorithms mostly useless.

Developing algorithms for such complex forest types requires access to good quality data with overlapping field inventories and remote sensing surveys. Of special interest to the problem of detection of individual trees and overall extraction of forest parameters from remote sensing data are machine learning and deep learning approaches. There is a lot of research on the topic that shows promising results^8–10^, but such approaches rely even more heavily on the availability of good quality reference data to be useful. In this article, we describe a dataset that we released openly in the hope to contribute to further facilitating the development of algorithms for detection of individual trees. All described data and an end-to-end implementation of a baseline detection algorithm and a procedure to match detected trees to ground truth data are freely available. The main inspiration for publishing this dataset is the NewFor Alpine benchmark open dataset^11^. It provides a similar collection of field inventory ground plots in the Alpine area covered by a UAV LiDAR survey. The main differences and similarities between our dataset and the NewFor benchmark are described below.

## Materials and methods

This section provides detailed descriptions of the dataset, a simple matching algorithm for evaluation of detection results, and a basic baseline approach for individual tree detection to benchmark against.

### Dataset

The dataset consists of a field inventory survey and overlapping UAV LiDAR point clouds and RGB orthophotos. The study area is located in Perm Krai, Russia, approximately 90 km to the east of the region’s administrative center, Perm. The forest in this area is mixed and dense, with complex and irregular canopy structure. The field survey consists of 3600 trees across 10 rectangular ground plots 100 m in length and 50 m in width. Every tree is represented as a point in the UTM 40N coordinate reference system (EPSG:32640). Every tree has a species label and diameter at breast height, measured with calipers at 1.3 m from the ground at two perpendicular directions and averaged. The inventory covers seven species of trees, approximately equally split among coniferous and deciduous: spruce, birch, fir, aspen, tilia, alder, and willow. Approximately 20% of the trees have height data measured during the inventory, and 10% have ages measured on core samples.

The LiDAR sensor used for the survey is AGM-MS3, with 640 kHz acquisition rate, 300-m range, and spatial accuracy of 3–5 cm. The mean height of data acquisition is 150 m, with the UAV configured to follow the terrain using the SRTM elevation map as a reference. The point clouds were preprocessed by removing duplicates and noise and classifying the points that represent the ground, which allows normalizing height within the point clouds. The duplicate removal was run with a threshold distance between points of 1 mm. Noise was removed by visually inspecting the point cloud and manually selecting height thresholds to cut off points that are lower than the ground or higher than the canopies. Height normalization allows treating the Z coordinate as height above ground rather than the absolute elevation, which simplifies many subsequent steps. The raw point clouds were processed with the combination of the AGM ScanWorks software from the sensor vendor and the TerraScan software. The average point density in the clouds is 37 points per square meter.

The camera used in the orthophoto survey is Sony A6000. The resolution of the RGB orthophoto is 7 cm per pixel.

The field inventory was conducted in May 2021, the UAV laser scanning and RGB surveys were conducted in July 2021, all in leaf-on conditions.

It is important to note that the locations of the trees recorded in the field survey are measured relatively close to the ground, while detection algorithms for UAV LiDAR point clouds usually detect the tops of the trees. There can be a significant distance in the XY plane between the location of the trunk at breast height and the highest point of the canopy because of tilt, complex canopy shape of broadleaf species, or both.

**Figure 1:**
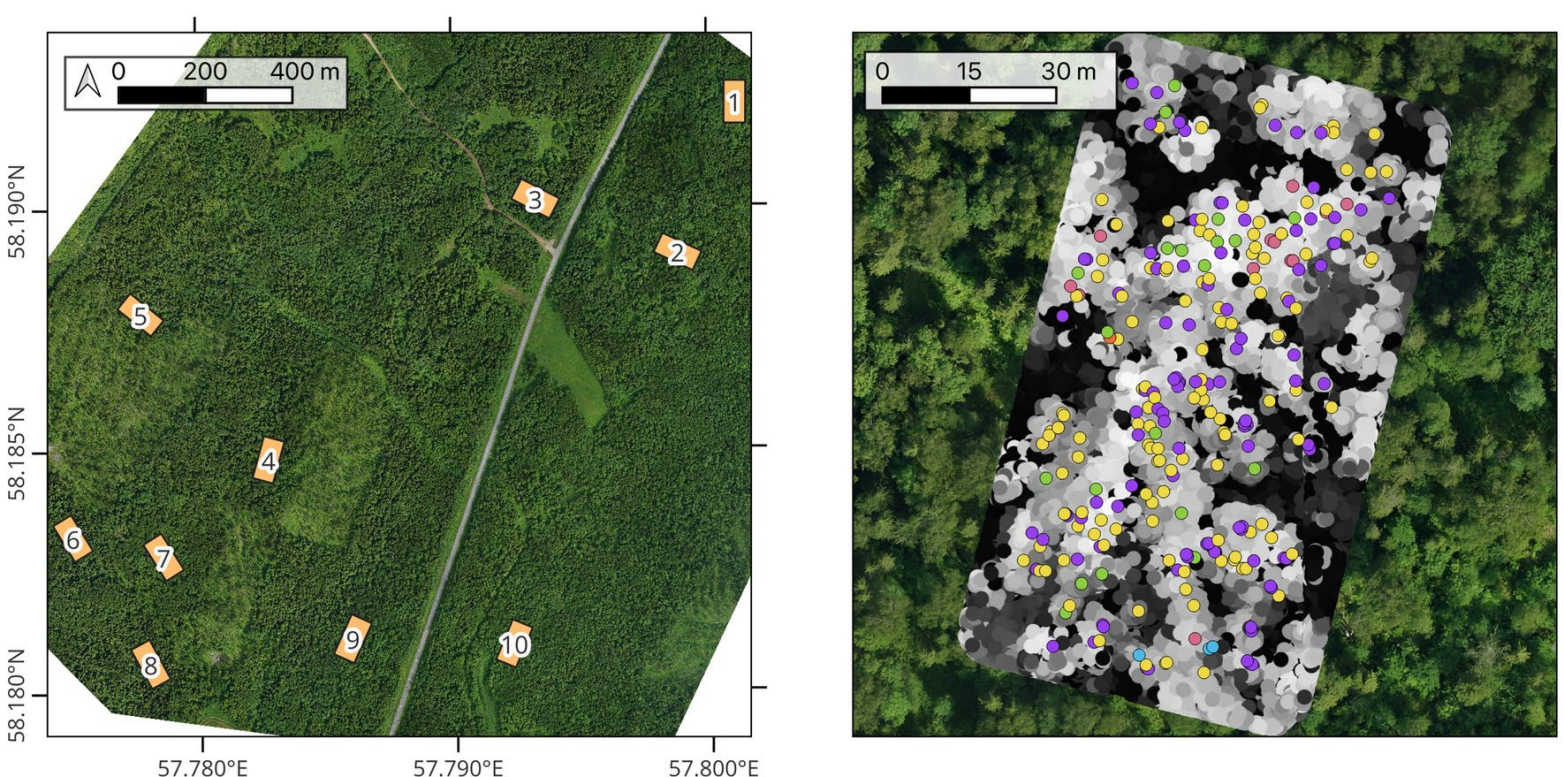
The study region and the data. Left: The locations of field survey plot boundaries on a full-size RGB orthophoto. Each plot is a 50 by 100 m rectangle, with every tree within measured and recorded. Buffered cutouts of the orthophoto come with the dataset, along with LiDAR point clouds. Right: A close up of plot number 4. Each colored point represents a single tree of a different species, on top of a point cloud colored by the value of the height of each point (lower points are dark, higher points are bright) and the same orthophoto.

**Figure 2:**
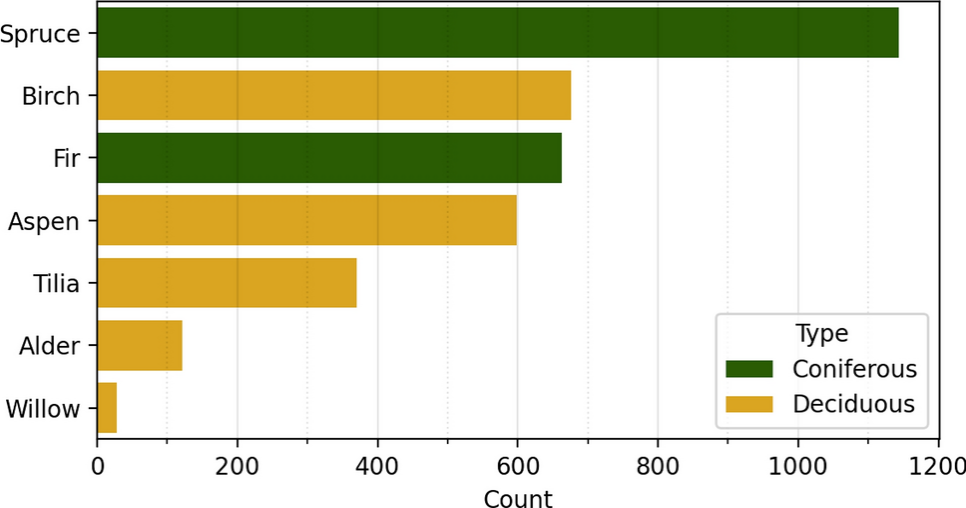
The distribution of tree species in the dataset. The dominant species is spruce. The data is split almost evenly among coniferous and deciduous species.

**Figure 3:**
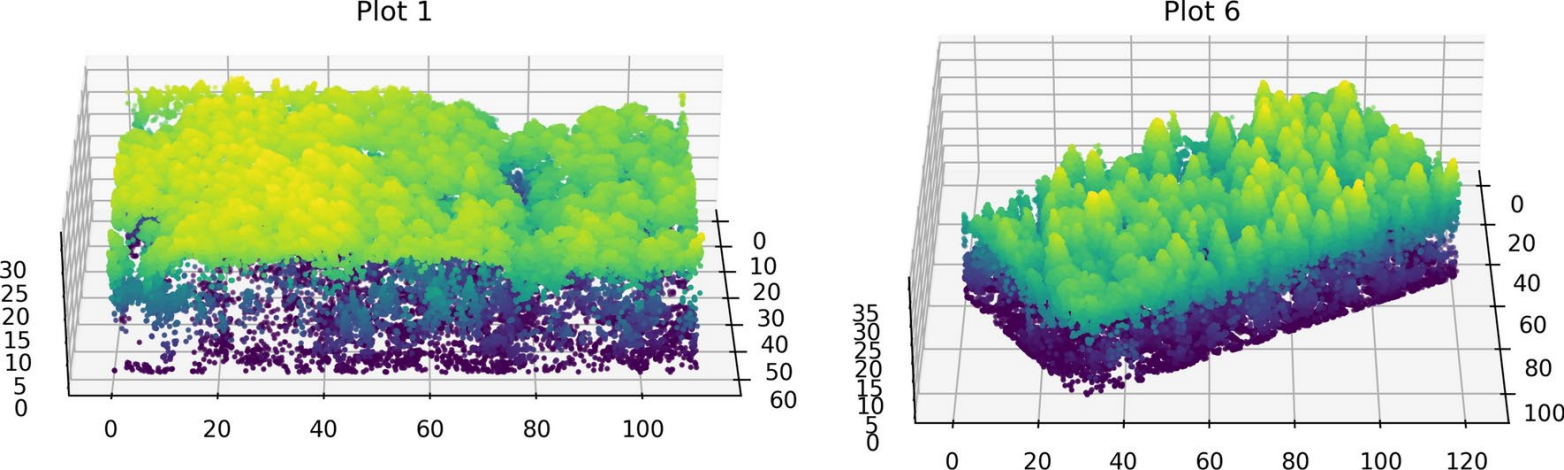
3D visualizations of point clouds from plots 1 and 6. Note the difference in the canopy structure: plot 1 is predominantly deciduous, while plot 6 is coniferous. There is no single local maxima filter window size that will produce satisfactory results for both of these plots at the same time.

Figure 1 shows the locations of the ground plots over the full size RGB orthophoto on the left and a visualization of the field inventory and a point cloud in 2D for a single plot on the right. Figure 2 shows the distribution of species in the dataset. The dominant species in the data is spruce, but overall the trees are evenly split between deciduous and coniferous species. Figure 3 shows two of the point clouds in 3D, highlighting the differences in canopy structure complexity between predominantly deciduous and coniferous plots.

#### Comparison to other datasets

Our dataset is in many ways similar to the NewFor benchmark^11^. It serves the same purpose and also offers overlapping field survey ground plots and UAV LiDAR point clouds. There are, however, many notable differences. The NewFor benchmark covers much more diverse regions, including ground plots from France, Italy, Switzerland, Austria, and Slovenia, while all our data comes from the same area. The tree species covered by the datasets are also different: both contain spruce and fir, but the NewFor data also has beech, Scots pine, larch, sycamore, and poplar, while ours also has birch, aspen, tilia, alder, and willow. Our dataset has more than twice as many individual trees as the Alpine benchmark, and the forest is denser, more complex, and more diverse, making it more complicated to detect trees in. Our data contains very mild terrain variations, while the slopes of the terrain in Alpine data are very steep. This plays a role during height normalization, since subtraction of steep terrain introduces artificial slope to the points in the canopy, changing the overall shape of the tree. The point clouds in our dataset are denser as well, making them more detailed, but more computationally heavy to work with. Our dataset has an additional information source—an RGB orthophoto that allows development of algorithms that fuse multimodal data, which, we believe, is a key to success in such complex environments. Our dataset has species labels for every surveyed tree, but only partial coverage of tree heights and no timber volume information at all.

Another similar dataset is the NeonTreeEvaluation Benchmark^12^, which offers bounding box annotation for tree detection across a wide range of different forest types. It offers coregistered RGB, LiDAR, and hyperspectral images over 31,000 individual trees. The main difference in the reference data between the NeonTreeEvaluation Benchmark and our dataset is the source: our data comes from a field inventory and thus has additional tree information that can be used in downstream tasks, such as species classification or timber volume prediction, while the NeonTreeEvaluation Benchmark annotations are created from the RGB photo and thus only offer the positions and sizes of trees.

Another dataset is the IDTReeS 2020 Competition Data^13^ aimed to develop algorithms for delineation and species classification of individual tree crowns in RGB, LiDAR, and hyperspectral data. It offers bounding box annotations for 1200 individual trees covered by RGB, LiDAR, and hyperspectral images in 3 national forests in the USA. Similarly, the source of the data is annotation of images, not a field inventory.

There are also datasets available that don’t have LiDAR point cloud coverage, or use terrestrial LiDAR instead of UAV LiDAR, or use photogrammetric point clouds instead of LiDAR point clouds. We do not mention them here because we specifically focus on UAV LiDAR.

**Table 1:** Ground plot statistics and baseline detection results. Point density is calculated as the number of points in the point cloud divided by the area of its 2D convex hull. Recall is the proportion of ground truth trees that have been detected, precision is the proportion of the detections that are actual ground truth trees, F1-score is the harmonic mean of the precision and recall. Distance is the average 2D distance between a detected tree and its match in the ground truth data.

Plot	Tree count	Dominant type	Point density	Recall	Precision	F1-score	Distance
1	420	Deciduous	31.7	0.69	0.45	0.54	0.73
2	365	Deciduous	47.9	0.48	0.56	0.52	0.79
3	332	Deciduous	40.3	0.62	0.40	0.48	1.08
4	261	Coniferous	33.5	0.56	0.38	0.46	1.02
5	208	Coniferous	14.2	0.65	0.36	0.45	1.02
6	290	Coniferous	39.1	0.61	0.36	0.45	0.80
7	408	Deciduous	41.9	0.59	0.45	0.51	0.89
8	341	Coniferous	35.5	0.65	0.39	0.48	0.82
9	459	Coniferous	42.1	0.48	0.59	0.53	0.98
10	518	Deciduous	42.9	0.38	0.75	0.51	0.78
			Average	0.57	0.48	0.50	0.89

**Table 2:** Matching results for a range of local maxima filter window sizes. The size of the window controls the tradeoff between precision and recall: smaller windows lead to many more detections, many of which are false positives, thus increasing recall and decreasing precision, while larger windows lead to selection of only the most dominant trees, leaving many undetected and thus decreasing recall and increasing precision. F1-score reflects this disbalance. The value of the second parameter, height threshold, has almost no effect on the results and is thus not included in the exploration.

Window size	Height threshold	Recall	Precision	F1-score	Distance
0.50	3.00	0.95	0.03	0.05	0.33
1.00	3.00	0.86	0.10	0.17	0.56
1.50	3.00	0.71	0.27	0.39	0.76
2.00	3.00	0.56	0.46	0.50	0.88
2.50	3.00	0.42	0.62	0.50	0.95
3.00	3.00	0.32	0.75	0.45	0.97
3.50	3.00	0.23	0.84	0.36	0.94
4.00	3.00	0.18	0.91	0.29	0.91
4.50	3.00	0.14	0.96	0.24	0.90
5.00	3.00	0.11	0.97	0.20	0.90

### Baseline: local maxima filtering

We provide an implementation of a very basic baseline algorithm for detection of individual trees within a point cloud: local maxima filter. It is the core of many other detection methods, which often vary only on what the local maxima filter is applied on, with what parameters, how many times, and how the results are merged into the final detection. The version we used is the most basic of all: it is a one pass filter directly on the point cloud and is parametrized by a fixed size window and a threshold for height that excludes all low points from consideration. Note that the algorithm assumes that the point clouds are height normalized and treats the Z coordinate as height above ground. Our implementation first discards the points lower than the height threshold. Then it constructs a kd-tree for all the points in the point cloud, and iterates through unprocessed points, repeatedly querying for neighbors in a sphere of the specified window size and marking all points but the highest as processed. Once it encounters a point that is the highest in its neighborhood, it marks it as a local maximum.

As mentioned, the algorithm can be improved in many ways, the easiest of which is a careful tuning of the window size for every plot. However, this approach is a priori not good enough to work on such complex data. Its main principle is very naive, and it does not fully utilize the vertical structure information available in the point cloud and does not use RGB data at all. The main purpose of providing it is to publish the dataset with an end-to-end example of implementing a detection procedure.

### Matching algorithm

To evaluate the results, an automated and deterministic method for matching detected trees to the ground truth data is required. It should determine which ground truth trees, if any, the detected candidates correspond to. It should also classify all the trees from both sets as either a true positive, meaning that the ground truth tree was successfully detected, a false negative, meaning that a ground truth tree was not detected, or a false positive, meaning a tree was detected when there is none.

We implemented a matching procedure that considers the locations and heights of the trees, and falls back to using only locations when the height information is not available for the ground truth tree. It is parametrized by the maximum allowed 2D distance and the maximum height difference between a detected and a ground truth tree to consider them a match. First, it constructs a distance matrix between the ground truth tree locations and the detected candidates and filters out the pairs for which the distance is larger than the allowed maximum. It then iterates over the remaining pairs in the order of increasing distance, and marks the pairs with suitable heights in which both trees are not yet assigned a class as a true positive. The pairs in which one of the trees is already assigned a class are marked as either a false positive or a false negative, and the pairs in which both trees are assigned a class are skipped. Finally, it marks all unmatched ground truth trees as false negatives, and all unmatched candidate trees as false positives.

**Figure 4:**
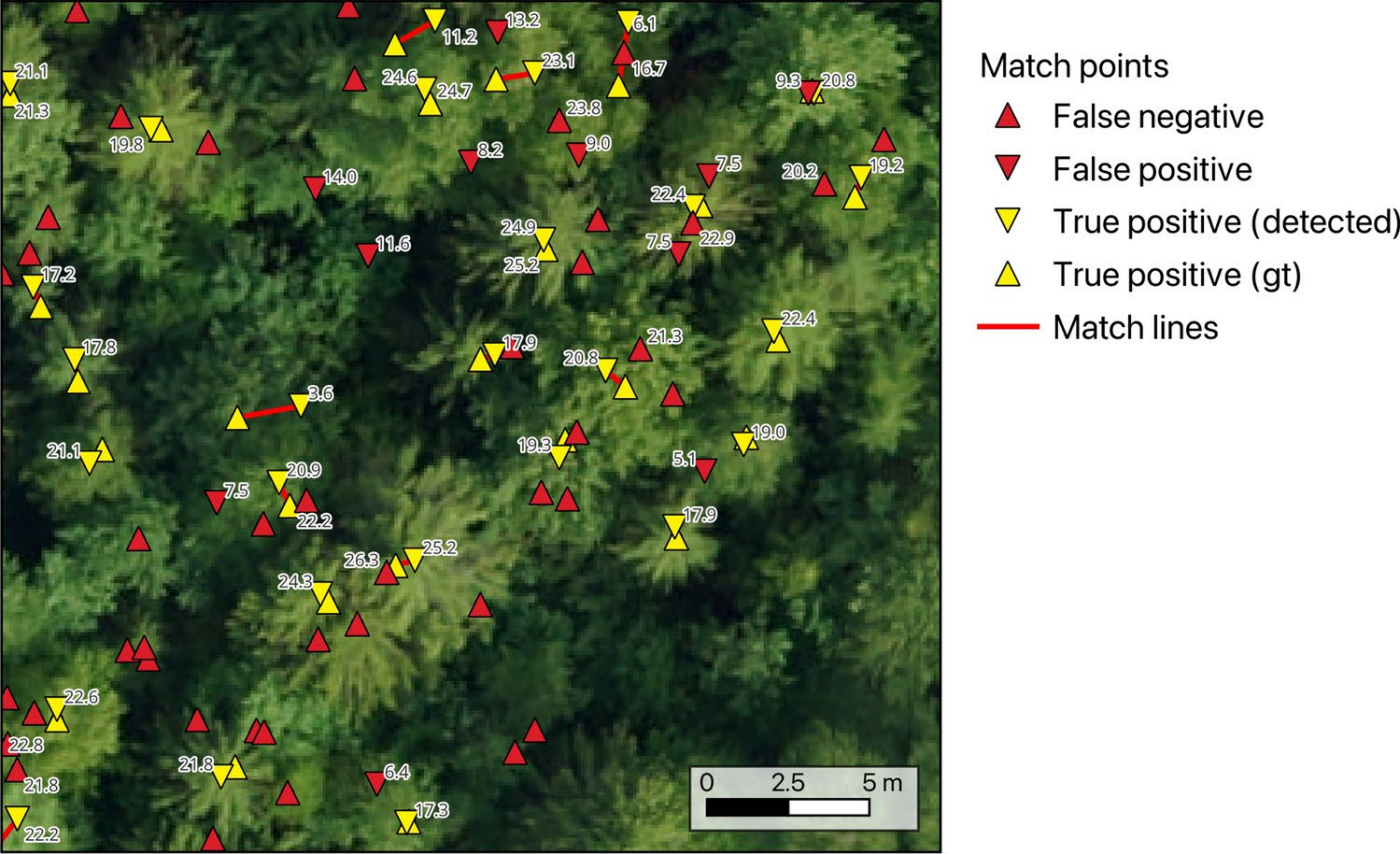
A fragment of plot number 10 with the visualization of detection and matching results. Triangles that point upwards are trees from the field survey, triangles that point downwards are candidates detected by the algorithm. Red triangles represent trees that have not been matched between the detection results and the ground truth, and yellow triangles connected by red lines represent matches. The numbers are the heights of the trees. The detection was run with a window size of 2 m and a height threshold of 3 m. The matching algorithm was run with a max distance of 5 m and a max height difference of 3 m.

An example of the detection and matching results is presented in Fig. 4. Each true positive is a pair of points, one from the ground truth and the other from detected candidates. They are represented in the figure as yellow triangles connected by red lines. Errors are represented by red triangles. Numbers near the triangles are heights—all the detected tree candidates have them, but only part of the ground truth data does.

## Results

Table 1 shows a summary of the main metrics achieved by the baseline algorithm for every plot, and Fig. 4 shows a fragment of its output processed by the matching algorithm. The metrics were achieved by running the local maxima filter with a window size of 2 m and a height threshold of 3 m. The matching algorithm was run with a maximum distance between matches of 5 m and a maximum height difference of 3 m. We report the metrics that are most commonly used to evaluate individual tree detection results. Recall is the proportion of ground truth trees that have been detected, it is the ratio of the number of true positives to the number of true positives and false negatives. Precision is the proportion of the detected trees that are successfully matched to ground truth trees, it is the ratio of the number of true positives to the number of true positives and false positives. F1-score aims to combine precision and recall into a single metric, it is the harmonic mean between them. Distance is the average distance between a detected tree and its corresponding ground truth tree. Note that the distance metric should be interpreted with care, since it mostly compares different points: the detection results in most algorithms correspond to the top of the canopy, while the ground truth point is the location of the trunk at breast height, so even perfect detection of every tree will result in non-zero distance error. Moreover, it is artificially limited from above by the parameters of the matching procedure.

The average values for all of these metrics are around 50%, meaning that approximately half of the trees in the ground truth data are detected correctly, and there are as many false positives as there are false negatives. These values vary greatly with the window size of the local maxima filter. Making a window smaller increases recall but decreases precision, since there are many more detections, many of which are false positives. Running the algorithm with a window size of 1 m results in average recall of 86%, but precision of only 10% and F1-score 17%. Similarly, increasing the window size drops recall but boosts precision. Running the algorithm with a window size of 5 m results in average precision of 97%, but recall only 11% and F1-score 20%. A more thorough exploration of this dependency is presented in Table 2, which reports metrics for a range of possible values of the window size.

Note in Fig. 3 how different are the canopy structures of plots with predominantly coniferous and deciduous trees. It is evident that using the same parameters for the local maxima detection in both types of forests will lead to suboptimal results, which further complicates the development of universal algorithms for such data.

As mentioned in the description of the local maxima filtering algorithm, we do not expect good performance of such a simple algorithm on data of this complexity. It does not fully utilize 3D structure information available in the point cloud and does not use the RGB data at all. It is also not a good fit for detecting broadleaf tree species, especially in dense forest stands, since their canopies are usually complex, often without a single highest point at the middle of the canopy, but many such points in irregular shapes. The main reason for including it at all is to release the dataset together with an end-to-end example of implementing and evaluating a detection algorithm. The code for generating the reported results is available alongside the dataset in the hope of making it easy to understand and start working with the data.

## Discussion

We present an open access dataset for development, evaluation, and comparison of algorithms for detection of individual trees within UAV LiDAR point clouds and RGB orthophotos in dense mixed forests. The dataset consists of a detailed field inventory of 3600 trees, with an even mix of deciduous and coniferous species, covered by a dense point cloud and a high resolution orthophoto. We released this dataset to be freely used for research purposes, along with implementations of a basic baseline detection algorithm and a matching procedure for evaluation. The data covers the type of forest for which simple algorithms cannot generate good enough results and which has been a target for research into more complex algorithms for a long time. Especially interesting is the possibility of developing algorithms that fuse multimodal data: active LiDAR point clouds and passive RGB images. Fusion of data from different sources is a reliable way to improve the quality of many tasks in remote sensing for forestry applications^14,15^. For forests that are as dense and complex as the one in the presented dataset, neither data source on its own has been enough to solve individual tree detection to an acceptable level of accuracy. We believe the most promising results will come from algorithms that utilize both, since they will have access to very different information about the same objects: color and texture from the RGB and 3D structure from the LiDAR. Even though the explored minimalistic baseline detection algorithm does not utilize the orthophoto in any way, the code that comes with the dataset has an example of a simple way to enrich the point cloud with color information by sampling the orthophoto. We hope that this dataset will be a useful addition to the field.

## Data Availability

The described data is openly available on Kaggle at kaggle.com/datasets/sentinel3734/tree-detection-lidar-rgb. Alongside it, there are several notebooks with example Python code that show how to load and visualize the data, as well as the implementations of the local maxima filtering and the matching algorithms described in the text. There are also many utility functions with examples of usage, for example, for exporting the results to GIS formats or normalizing height of a point cloud with classified ground. The notebooks can be run directly on Kaggle through a web browser.
